# Infection of *Ophiocordyceps sinensis* Fungus Causes Dramatic Changes in the Microbiota of Its *Thitarodes* Host

**DOI:** 10.3389/fmicb.2020.577268

**Published:** 2020-12-03

**Authors:** Hua Wu, Zhong-Chen Rao, Li Cao, Patrick De Clercq, Ri-Chou Han

**Affiliations:** ^1^Department of Plants and Crops, Faculty of Bioscience Engineering, Ghent University, Ghent, Belgium; ^2^Guangdong Key Laboratory of Animal Conservation and Resource Utilization, Guangdong Public Laboratory of Wild Animal Conservation and Utilization, Institute of Zoology, Guangdong Academy of Sciences, Guangzhou, China

**Keywords:** Chinese cordyceps, *Ophiocordyceps sinensis* fungus, *Thitarodes/Hepialus* moth, microbiota, hemolymph, gut

## Abstract

The Chinese cordyceps is a unique and valuable parasitic complex of *Thitarodes*/*Hepialus* ghost moths and the *Ophiocordyceps sinensis* fungus for medicine and health foods from the Tibetan Plateau. During artificial cultivation of Chinese cordyceps, the induction of blastospores into hyphae is a prerequisite for mummification of the infected *Thitarodes* larvae. To explore the microbial involvement in the induction of mycelia-blastospore transition, the microbiota of the hemolymph and gut from *Thitarodes xiaojinensis* larvae with or without injected *O. sinensis* blastospores were investigated by culture-dependent and -independent methods. Twenty-five culturable bacterial species and 14 fungal species, together with 537 bacterial operational taxonomic units (OTUs) and 218 fungal OTUs, were identified from the hemolymph and gut of samples from five stages including living larvae without injected fungi (A) or with high blastospore load (B), mummifying larvae without mycelia coating (C), freshly mummifying larvae coated with mycelia (D), and completely mummified larvae with mycelia (E). Two culturable bacterial species (*Serratia plymuthica, Serratia proteamaculans*), and 47 bacterial and 15 fungal OTUs were considered as shared species. The uninfected larval hemolymph contained 13 culturable bacterial species but no fungal species, together with 164 bacterial and 73 fungal OTUs. To our knowledge, this is the first study to detect large bacterial communities from the hemolymph of healthy insect larvae. When the living larvae contained high blastospore load, the culturable bacterial community was sharply inhibited in the hemolymph but the bacterial and fungal community greatly increased in the gut. In general, high blastospore load increased bacterial diversity but sharply decreased fungal diversity in the hemolymph and gut by OTUs. The bacterial loads of four culturable species (*Chryseobacterium* sp., *Pseudomonas fragi*, *S. plymuthica, S. proteamaculans*) increased significantly and *O*. *sinensis* and *Pseudomonas* spp. became dominant microbes, when the infected larvae became mummified, indicating their possible involvement in the larval mummification process. The discovery of many opportunistic pathogenic bacteria in the hemolymph of the healthy larvae, the larval microbial diversity influenced by *O. sinensis* challenge and the involvement of dominant bacteria during larval mummification process provide new insight into the infection and mummification mechanisms of *O. sinensis* in its *Thitarodes* hosts.

## Introduction

The Chinese cordyceps, a unique parasitic complex formed by the parasitism of *Thitarodes*/*Hepialus* spp. (Hepialidae, Lepidoptera) by the *Ophiocordyceps sinensis* fungus in the Qinghai-Tibet Plateau, is a highly valuable biological resource for medicines and health foods in China but also elsewhere in the world (Yue et al., 2013; Li et al., 2016; Baral, 2017; Pouliota et al., 2018; Qin et al., 2018). Diverse bacteria and fungi in the environment and in the *Thitarodes*/*Hepialus* hosts may influence the formation of the Chinese cordyceps.

Microbial communities of the wild Chinese cordyceps have been extensively investigated using traditional culture-dependent (Zhang et al., 2010) and culture-independent methods (Liang et al., 2008; Zhang et al., 2009, 2010; Xia et al., 2015, 2016). The microbiota of the wild Chinese cordyceps and its microhabitats are highly diverse. More than 22 species belonging to 13 genera as anamorphs have been reported in wild Chinese cordyceps specimens (Jiang and Yao, 2003). Approximately 600 isolates were obtained by culture-dependent methods from different parts of the Chinese cordyceps (including stromata, sclerotia, and external mycelial cortices) and its soil microhabitats (Zhang et al., 2010). The microbiota of three different sections (stromata, sclerotia, and mycelial cortices) from wild Chinese cordyceps specimens contained 572 fungal strains and 92 putative operational taxonomic units (OTUs) by a culture-dependent method, and 118 putative OTUs by a culture-independent method (Zhang et al., 2010). A high diversity of fungal communities, with the dominant fungal phylum Ascomycota (such as *Ophiocordyceps*, *Verticillium*, *Pseudallescheria*, *Candida*, and *Ilyonectria*), inhabiting the Chinese cordyceps and its microhabitats was revealed by using Illumina high-throughput sequencing (Xia et al., 2016). High throughput sequence analysis of 16S rRNA genes and ITS regions showed that the main bacterial groups were Proteobacteria, Acidobacteria, Bacteroidetes, Actinobacteria, and Firmicutes, while the Ascomycota, Basidiomycota, and Zygomycota were the main fungal phyla in wild Chinese cordyceps (Xia et al., 2016). Bacterial diversity grouped into 23 phyla including Proteobacteria, Actinobacteria, Acidobacteria, and Verrucomicrobia, in the soils of the native habitats of the Chinese cordyceps was investigated using Illumina sequencing data (Yang et al., 2015). Apart from *O. sinensis*, *Paecilomyces sinensis* (Chen et al., 1984), *Mortierella hepiali*, *Scytalidium hepiali* (Li, 1988), *Tolypocladium sinensis*, *Cephalosporium acremonium*, *Paecilomyces hepiali* (Dai et al., 2017), *Penicillium chrysogenum*, and *Pseudogymnoascus roseus* were also found from wild Chinese cordyceps (Jiang and Yao, 2003; Zhang et al., 2010).

Insects depend to a degree on the diverse communities of microbiota in their gut for basic functions, such as nutrition, protection from parasites and pathogens, modulation of immune responses, communication and reproduction (Engel and Moran, 2013; Kwong and Nancy, 2016; Wei et al., 2017). Zhuo et al. (2004) isolated 8 bacterial genera (*Staphylococcus*, *Bacillus*, *Klebsiella*, *Pseudomonas*, *Aeromonas*, *Plesiomonas*, *Sporosarcina*, and *Neisseria*) from the guts of wild *Thitarodes gonggaensis* larvae, with *Staphylococcus* being dominant. Liu et al. (2008) also isolated eight bacterial genera (*Enterobacter*, *Carnobacterium*, *Novosphingobium*, *Acinetobacter*, *Pseudomonas*, *Klebsiella*, *Pantoea*, and *Delftia* also from the guts of wild *T. gonggaensis* larvae with dominant *Enterobacter* bacteria, based on culture-dependent and -independent methods. Yu et al. (2008) obtained several fungal genera (*Mortierella*, *Trichosporon*, *Mucor*, *Rhinocladiella*, *Cephalosporium*, *Rhodiola*, and *Mastigobasidium*) from the guts of the same ghost moth species by RFLP analysis, and *Cryptococcus magnus*, *Geomyces* sp., and *Trichosporon porosum* by culture method. In a study of the internal microbial community in unfertilized eggs of *Thitarodes pui*, 348 bacterial genera (dominant genera included *Wolbachia*, *Spiroplasma*, *Carnobacterium*, *Sphingobium*, and *Acinetobacter*) belonging to 26 phyla, 58 classes, 84 orders, and 120 families were identified from 1,294 OTUs; 289 fungal genera, mainly including *Aureobasidium*, *Candida*, and *Cryptococcus*, were identified, and they belonged to five phyla (Ascomycota, Basidiomycota, Chytridiomycota, Glomeromycota, and Zygomycota), 26 classes, 82 orders, and 165 families (Liang et al., 2019). Although different bacterial or fungal communities were isolated even from the guts of the same wild *Thitarodes* species living in different locations, the functions of these microbes in the association with the host development were totally unknown. No microbes were also reported from the hemolymph of *Thitarodes/Hepialus* spp. larvae so far.

To protect this natural resource, artificial cultivation of *O. sinensis* fruiting bodies on rice media (Cao et al., 2015) and culturing of the host caterpillar *Thitarodes* sp. on artificial media (Cao and Han, 2014; Tao et al., 2016) has been successfully established in low-altitude laboratories mimicking environmental conditions of the wild habitat. After being introduced into the hemolymph of the larvae, the spindle blastospores may produce more spindle cells by budding growth and/or grow into elongate hyphal bodies (pseudohyphae) and hyphae by apical growth under the induction of some unknown factors (Liu et al., 2019, 2020), as reported in the dimorphic fungi *Candida albicans* and *Ustilago maydis* (Ruiz-Herrera et al., 2020) and the entomopathogenic fungus *Metarhizium rileyi* (Boucias et al., 2016). However, the slow mummification of the *Thitarodes* larvae post infection is still an unresolved problem and an obstacle for commercial production of Chinese cordyceps (Li et al., 2016; Qin et al., 2018; Han et al., 2019). The living infected larvae might harbor the spindle blastospores in the hemolymph for several months, contrary to other entomopathogenic fungi such as *Metarhizium anisopliae* and *Beauveria bassiana* which cause the death of their host larvae within a few days (Meng et al., 2015; Rao et al., 2019).

The ability of pathogenic fungi to switch between yeast or spindle cells (blastospores) and hyphae is well-studied in plant pathogens such as *U. maydis* (Gauthier, 2015) and the human prevalent fungal pathogen *C. albicans* (Desai, 2018). This dimorphism is correlated with pathogenicity along with the infection process (Nemecek et al., 2006; Gauthier, 2015). *O. sinensis* also exhibits spindle cell-hyphae dimorphism (Liu et al., 2019, 2020). In other fungal systems, a number of external inputs such as nutrients, temperature, pH, CO_2_ and chemicals and quorum sensing molecules for the morphological transition in dimorphic fungi have been identified (Gauthier, 2015; Zhu et al., 2017). Inducers responsible for hyphal conversion from blastospores in *O. sinensis* and for the mummification of the infected larvae are unclear.

To explore the possible involvement of the microbial communities in the induction of mycelial transition from blastospores, the microbiota of the hemolymph and gut from the larvae of *T. xiaojinensis*, a highly potential ghost moth species for commercial cultivation, with or without injected *O. sinensis* blastospores were investigated by culture-dependent and culture-independent methods.

## Materials and Methods

### Insects

*Thitarodes xiaojinensis* was reared according to the methods described by Tao et al. (2016) and Liu et al. (2019). Briefly, the insect pupae were collected from 3500–4000 m altitude mountains in Xiaojin, Sichuan Province, China, and housed in plastic containers (*L* = 50 cm; *W* = 40 cm; *H* = 30 cm) with moist moss kept at 9–17°C and 50–80% relative humidity. The emerged adults were housed in equal proportions of males and females in small mosquito nets (*L* = 104 cm; *W* = 50 cm; *H* = 50 cm) for mating and oviposition. The hatched larvae were transferred to a culture room at 9–13°C in Guangzhou, Guangdong, China, and offered the roots of *Potentilla anserina* as food to obtain 5th instar larvae (average fresh weight = 0.52 ± 0.03 g) for fungal infection. The insect species was identified as *T. xiaojinensis* by using the amplified Cytochrome b sequence with the primers CB1 (TATGTACTACCATGAGGACAAATATC) and CB2 (ATTACACCTCCTAATTTATTAGGAAT (Zou et al., 2011; Tao et al., 2016).

### Fungal Isolate

The KD1223 isolate of *O. sinensis* isolated from the fruiting bodies of wild *O. sinensis* in Sichuan, China was cultured on PPDA medium (liquid PPDA medium: 200 g potato extract, 20 g glucose, 10 g peptone, 1.5 g KH_2_PO_4_, 0.5 g MgSO_4_, 20 mg vitamin B_1_, and 1,000 ml distilled water; solid PPDA medium: 15% agar in liquid PPDA medium) at 13°C. The fungal isolate was identified by using the amplified sequence from the internal transcribed spacer (ITS; ITS1-5.8S-ITS2) of the nuclear ribosomal DNA as described by Cao et al. (2015). The identified *O. sinensis* isolate was preserved at −80°C in the Guangdong Institute of Applied Biological Resources, Guangzhou, China.

The fungal colonies cultured on the PPDA plates at 13°C for 60 days were transferred to 250 mL flasks containing 150 mL liquid PM medium (200 g potato extract, 20 g maltose, 10 g peptone, 1.5 g KH_2_PO_4_, 0.5 g MgSO_4_, 20 mg vitamin B_1_, and 1,000 mL distilled water; Liu et al., 2019). The flasks were incubated at 13°C on a 120 rpm rotary shaker, and after 50 days the blastospores from the flasks were harvested by using three layers of sterile lens papers to remove hyphae and large particles, then the filtered solution was centrifuged at 8,000 rpm for 15 min at 10°C and the supernatant discarded. Harvested blastospores were re-suspended in sterile phosphate-buffered saline (PBS; pH 7.0) at a concentration of 3.0 × 10^6^ blastospores per mL. The blastospore suspension was kept at 4°C for less than 3 days before use for larval inoculation.

### Larval Infection

An aliquot of 4 μL suspension containing 1.2 × 10^4^ blastospores was injected into each 5th instar larva by a microinjection system (IM-31; Narishige, Japan). 150 larvae were used for each replicate and three replicates were set for each injection. Larvae injected with PBS buffer or without any injection were set as controls. The injected larvae were reared at 4°C for 1 week and then transferred to a culture room at 13°C. After 90 days, about 10 μL hemolymph of each injected larva (6th instar) was sampled for confirming the presence of the blastospores.

### Culture-Dependent Microbial Community

**FIGURE 1:**
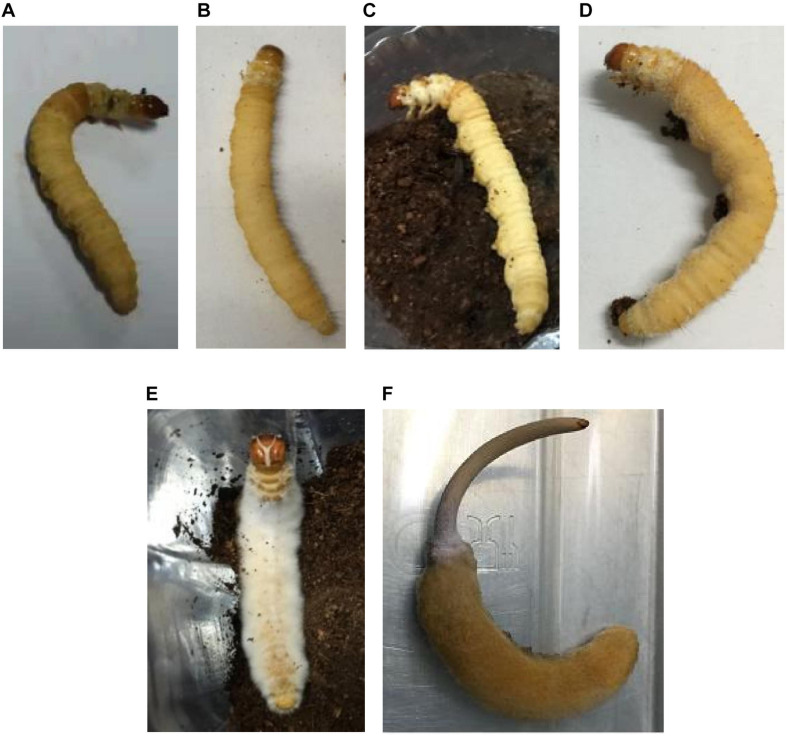
*Thitarodes* larvae with or without *Ophiocordyceps sinensis* infection. Living larvae without injected fungi **(A)**, living larvae with high blastospore load **(B)**, mummifying larvae not coated with mycelia **(C)**, freshly mummifying larvae coated with mycelia **(D)**, completely mummified larvae with mycelia **(E)**, and Chinese cordyceps **(F)**.

For investigation of the culture-dependent microbial community, after 90 days post injection, the hemolymph (100 μL from each larva; H) and guts (whole gut of each larva; G) of uninjected living larvae (A), and of injected larvae with fungal growth in four stages, including living larvae with a high blastospore load (B; 4.2 × 10^3^ blastospores per mL in the hemolymph), and freshly mummifying larvae without mycelia coating (C) were sampled, using micro-needles (pulled glass capillaries G-1 by a micropipette PC-10 puller, Narishige, Japan). The tissues of the mummified larvae coated with mycelia (D) and completely mummified larvae with mycelia (E; Figure 1) were sampled after the mycelial coating and cuticle of the larvae were carefully removed with sterile scalpels. All these samples were immediately placed in sterile centrifuge tubes with sterile PBS and divided into two parts: one for the culture-dependent method and another for the culture-independent method. AH, BH, and CH represented the hemolymph samples from Group A, B, and C, respectively; AG, BG, and CG represented the gut samples from Group A, B, and C, respectively; DT and ET refer to tissue samples from Group D and E. Thus, eight types of samples were collected for the analysis. For the process of hemolymph collection, the methods described by Clark (1982) and Herren et al. (2014) were employed with minor improvements. Briefly, under sterile conditions in a cabinet, the larvae from the laboratory culture (not from the wild) were washed three times with sterile distilled water, disinfected two times with 75% alcohol for 30 s, and rinsed three times with sterile distilled water. The larvae were dried on sterile filter paper before the hemolymph was carefully collected by sterile capillary micro-needles. For each sample type, three replicates containing three larvae per replicate were established.

The suspension was used to isolate the fungi and bacteria on the plates of LB (Lucia-Bertani), HIA (Heart infusion agar; BD, United States), G5 (PPDA supplemented with 0.5% milled fresh 6th instar *Galleria mellonella* larvae) and GSA (Gause’s synthetic agar; HKM, China), by the dilution method. The pH values of all these media ranged from 6.0 to 7.0. The plates were sealed with parafilm (BEMIS, United States) and cultured in the dark in incubators with or without aeration, at 13 and 23°C, respectively. Twelve plates were set up for each treatment.

After the bacteria and fungi appeared on the plates, they were individually collected to inoculate new plates until 30 days. Microbe isolates were first grouped according to their colony and morphological characteristics on LB or PDA plates. The resulting colonies were cultured in liquid LB (for bacteria) or PDA (for fungi) and kept at -80°C with 15% glycerol.

Genomic DNA of each isolate within each group was extracted with a simple and rapid method for fungi (Zhang et al., 2010) and a routine method for bacteria (Cao et al., 2015), and the V4 region of bacterial 16S rDNA and the ITS2 region of the fungal ITS gene were specifically amplified using PCR. Primers 27F (5′-AGAGTTTGATCCTGGCTCAG-3′) and 1492R (5′-TACGGYTACCTTGTTACGACTT-3′), and ITS1 (5′–TCCGTAGGTGAACCTGCGG-3′) and ITS4 (5′-TCCTCCGCTTATTGATATGC-3′) were used specifically for bacterial and fungal fragments, respectively. The polymerase chain reaction (PCR) mixture contained 5 μL of 10 × Pfu buffer, 4 μL of dNTP mixture (2.5 mM), 1 μL of each primer (10 μM), 2 μL of deionized formamide, 1 μL of MgCl2 (25 mM), 1 μL of genomic DNA, and 0.5 μL of Pfu DNA polymerase in a total volume of 50 μL. PCR amplification was performed in a TGradient thermocycler (Applied Biosystems). For 16S rDNA and ITS, the mixture was heated for 30 s at 98°C, then subjected to 35 cycles of 10 s at 98°C, 10 s at 55°C, and 30 s at 72°C, and a final 10-min elongation step at 72°C. After confirmation of the PCR products by agarose gel electrophoresis, the fragments were cleaned using a MinElute PCR Purification Kit (Qiagen, Germany). The purified PCR products then were sequenced by Sangon Biological Engineering (Shanghai) Co., Ltd. The resulting sequences were compared with the data set in NCBI GenBank.

The sequences of one representative isolate or clone for each bacterial and fungal species were submitted to GenBank^1^ and assigned accession numbers (MT626045-MT626058, MT631975-MT631999) for the sequences of cultivated isolates and clones, respectively.

### Culture-Independent Community

Amplicon sequencing was employed to investigate the bacterial and fungal microbiota of the hemolymph and gut from the eight sample types, according to Liang et al. (2019). Briefly, total DNA of the hemolymph and gut samples after being ground in liquid nitrogen was extracted and purified by the MO BIO PowerSoil DNA Isolation Kit (MO BIO Laboratories, Inc., Carlsbad, CA, United States) following the instructions of the manufacturer. The purified DNA was quantified by the NanoDrop ND-3300 spectrophotometer (NanoDrop Technologies, Thermo Scientific, Wilmington, DE, United States). The V4 region of bacterial 16S rDNA with primers 515F/806R and the ITS2 region of the fungal ITS gene with primers ITS3/ITS4 were specifically amplified using PCR. The primers contained a 12-bp barcode sequence at the 5′-end to distinguish the samples. All the PCR reagents were purchased from TaKaRa, Dalian, China. The PCR reaction mixture (50 μL) contained Ex Taq DNA polymerase (0.5 units), 1 × Ex Taq loading buffer (10 μL), dNTPs (8 μL), 2 μL of each primer (10 mM), and DNA template (10–100 ng). PCR was performed by the ABI Gene Amp^®^ 9700 PCR System (Applied Biosystems, Waltham, MA, United States) with the parameters for bacterial-specific fragments: 95°C for 3 min; 35 cycles of 94°C for 30 s, 55°C for 1 min, and 72°C for 1 min; and 72°C for 10 min. For fungal-specific fragments, the PCR procedures included 95°C for 5 min; 30 cycles of 95°C for 30 s, 52°C for 30 s, and 72°C for 45 s; and final elongation at 72°C for 10 min. The amplifications were conducted in triplicate for each sample and the products were pooled to minimize the PCR bias. After evaluation by 2% agarose gel electrophoresis, PCR products were mixed in equidensity ratios according to the GeneTools Analysis Software (Version4.03.05.0, SynGene). The mixed products were purified with the EZNA Gel Extraction Kit (Omega Bio-Tek, Norcross, GA, United States).

Sequencing libraries with index codes were constructed by NEBNext Ultra^TM^ DNA Library Prep Kit for Illumina (New England Biolabs, MA, United States) according to the instructions of the manufacturer. After assessment on the Qubit 2.0 Fluorometer (Thermo Fisher Scientific, MA, United States) and Agilent Bioanalyzer 2100 system (Agilent Technologies, Waldbron, Germany), the libraries were sequenced on an Illumina Novo6000 PE250 platform, and 250 bp paired-end reads were generated (Berry Genomics Co., Ltd., Beijing, China). To obtain high-quality clean reads, paired-end raw reads were filtered according to the Trimmomatic (V0.33) quality control process (Bolger et al., 2014). Paired-end clean reads were merged using FLASH (v1.2.11) according to the relationship of the overlap between the paired-end reads (Magoè and Salzberg, 2011). Raw Tags were merged when at least 10 of the reads overlapped the read generated from the opposite end of the same DNA fragment, with the maximum allowable error ratio of the overlap region of 0.1. Sequences were assigned to each sample based on their unique barcodes and primers by Mothur software (V1.35.1; Schloss et al., 2009). Clean Tags were generated after barcodes and primers were removed.

Usearch software (V10; Edgar, 2010) was used to perform sequences analysis, and sequences with ≥ 97% similarity were assigned to the same OTUs (Tindall et al., 2010). The most frequently occurring sequence was extracted as the representative sequence for each OTU and was screened for further annotation. The obtained 16S and ITS amplicon data were submitted to sequence read archive (SRA) and assigned accession numbers PRJNA631104 and PRJNA631325, respectively.

For each representative sequence, the taxonomic classification (setting the confidence threshold default to ≥ 0.5) was carried out by SILVA (v. 119^2^) for bacteria and UNITE (v. 7.0^3^) for fungi. The OTU and its tags were removed when annotated as chloroplasts or mitochondria (16S amplicons) and not to the kingdom level, then the OTU taxonomy synthesis information tables for the final analysis were generated.

Based on the OTU table, the unique and the shared OTUs among the eight sample types in UpSetR plot were illustrated with UpSetR package in R software (Conway et al., 2017). The annotation ratio on each classification level was calculated to obtain the sequence composition of each sample at each classification level. Based on the relative abundance of species at each classification, the histogram was drawn with the ggplot2 package in R software.

For alpha-diversity and beta-diversity analyses, 294753 (A), 302110 (B), 275154 (C), 144858 (D) and 136746 (E) 16S rRNA tags per sample, and 219394 (A), 254271 (B), 271636 (C), 126816 (D) and 136673 (E) ITS tags per sample in OTU tables were used. QIIME (V1.9.1; Caporaso et al., 2010) was used to calculate bacterial and fungal alpha-diversity indices, including Chao1 and Simpson, to analyze the complexity of species diversity for a sample. Chao1 was selected to identify community richness. Simpson was used to identify community diversity. Beta-diversity analysis was used to evaluate differences of samples in species complexity. Non-metric multidimensional scaling (NMDS) based on Bray–Curtis distance matrixes was used for multivariate analysis of all samples, which was performed by Vegan package of R software. Based on the OTU table, two non-parametric analyses including analysis of similarity (ANOSIM) and non-parametric multivariate analysis of variance (Adonis) using distance matrices were performed by R software, to display the extent of differences among samples and to test whether the differences were significant (*p* < 0.05). A linear discriminant analysis (LDA) effect size (LEfSe) algorithm was employed to identify the taxa in different abundances (biomarkers; Segata et al., 2011) among samples. The effect size threshold of the LDA score was set to 2.

## Results

### Culture-Dependent Communities

Overall, 25 bacterial species belonging to 16 genera and 14 fungal species belonging to 13 genera were identified from the hemolymph, gut and tissues of 8 types of samples from 5 infection stages (Figure 1). Two bacterial species (*S. plymuthica* and *S. proteamaculans*) were shared and *Pseudomonas* spp. were dominant in the larval hemolymph and gut in all samples (Table 1). Interestingly, hemolymph from the healthy larvae also contained as many bacterial species as did the guts.

**TABLE 1:** Bacterial and fungal species identified in the larval hemolymph, gut and tissue by culture-dependent method.

	Microbe Species	A		B		C		D	E
		AH	AG	BH	BG	CH	CG	DT	ET
Bacteria	*Arthrobacter* sp.	**+**	**+**		**+**				
	*Carnobacterium maltaromaticum*	**+**	**++++**		**+**				
	*Chryseobacterium* sp.		**+**	**++**	**+**	**+**	**++**	**+++**	**++**
	*Enterobacterales*	**+**				**+**	**+**		**+**
	*Flavobacterium frigidimaris*					**+**	**+**	**+**	**++**
	*Kaistia terrae*				**+**				
	*Microbacterium* sp.	**+**	**+**		**+**				**+**
	*Micrococcaceae*		**+**		**+**				
	*Mycobacteroides salmoniphilum*				**+**				
	*Nocardiaceae*	**+**	**+**		**+**				
	*Pseudomonas brenneri*				**+**	**+**		**+**	
	*Pseudomonas fragi*	**+**	**+**		**++**	**++**	**++++**	**++++**	**++++**
	*Pseudomonas migulae*	**+**	**+**		**+**	**+**		**++**	**+**
	*Pseudomonas mohnii*		**+**						
	*Pseudomonas poae*					**+**	**++**		
	*Pseudomonas* sp.	**+**			**+**	**+**			**+**
	*Rahnella aquatilis*	**+**	**+**		**+**				
	*Rhizobium* sp.		**+**						
	*Rhodococcus* sp.	**+**	**+**		**+**				
	*Serratia plymuthica*	**+**	**+**	**+**	**+**	**+**	**++++**	**++**	**+++**
	*Serratia proteamaculans*	**+**	**+**	**+**	**++**	**++++**	**++**	**++++**	**++++**
	*Sphingobacterium kitahiroshimense*					**+**			
	*Stenotrophomonas rhizophila*	**+**		**+**					
	*Streptomyces* sp.				**+**				
	*Tsukamurella strandjordii*	**+**		**+**	**+**	**+**	**+**	**+**	
Fungi	*Apiotrichum porosum*		**+**		**+**				
	*Arthrinium* sp.						**+**		
	*Cladosporium halotolerans*				**+**				
	*Mucor hiemalis*								**+**
	*Mucor racemosus*				**+**				
	*Ophiocordyceps sinensis*					**++**			
	*Paecilomyces hepiali*							**+**	
	*Parengyodontium album*							**+**	
	*Penicillium commune*				**+**				
	*Phialemonium inflatum*		**+**		**+**				
	*Pseudogymnoascus* sp.				**+**			**+**	
	*Stachybotrys chartarum*				**+**				
	*Sterigmatomyces halophilus*		**+**						
	*Toxicocladosporium irritans*				**+**				
	**Bacterial species**	**14**	**14**	**5**	**18**	**12**	**8**	**8**	**9**
	**Fungal species**	**0**	**3**	**0**	**8**	**1**	**1**	**3**	**1**

**AH and AG, hemolymph and gut of un-injected living larvae; BH and BG, hemolymph and gut of living larvae with a high load of blastospores; CH and CG, freshly mummifying larvae without mycelia coating; DT, tissues of the mummified larvae coated with mycelia; ET, tissues of the completely mummified larvae with mycelia. +, ≤ 100 colonies detected in the plates; ++, > 100 ≤ 1,000 colonies detected in the plates; +++, > 1,000 ≤ 3000 colonies detected in the plates; ++++, > 3000 colonies detected in the plates*.*

As to the larval hemolymph, no fungi except the injected *O. sinensis* were detected from the agar plates. The detected bacterial isolates decreased sharply from 14 species in the living larvae without blastospores (AH) to 5 species in the living larvae containing a high load of blastospores (BH). *Arthrobacter* sp., *Carnobacterium maltaromaticum*, *Enterobacterales*, *Micro bacterium* sp., *Nocardiaceae*, *Pseudomonas fragi*, *Pseudomonas migulae*, *Pseudomonas* sp., *Rahnella aquatilis* and *Rhodococcus* sp., which were present in stage A, disappeared from the hemolymph in stage B (Table 1). It appeared that the bacterial community in the hemolymph was inhibited when the larvae were full of blastospores. Surprisingly, it was difficult to isolate *O. sinensis* by the tested media from the larvae even in the stage when they were filled with blastospores.

More colonies of *C. maltaromaticum* were detected from the gut of healthy larvae, and growing blastospores apparently stimulated the occurrence of fungal and bacterial species in the gut of the living larvae filled with blastospores (BG; Table 1). Three more bacterial species and 6 more fungal species including fungal species not detected from the healthy larvae were isolated from guts. Compared with AG, six bacterial species (*Kaistia terrae*, *Mycobacteroides salmoniphilum*, *Pseudomonas brenneri*, *Pseudomonas* sp., *Streptomyces* sp., and *Tsukamurella strandjordii*) and six fungal species (*Cladosporium halotolerans*, *Mucor racemosus*, *Penicillium commune*, *Pseudogymnoascus* sp., *Stachybotrys chartarum*, and *Toxicocladosporium irritans* were detected only from BG.

Interestingly, when the infected larvae (C, D and E) became mummified, four bacterial species including *Chryseobacterium* sp., *Pseudomonas fragi*, *S. plymuthica*, and *S. proteamaculans* strongly increased, whereas the fungal community in the gut sharply decreased (Table 1).

*Flavobacterium frigidimaris* was unique for freshly or completely mummified larvae. *Pseudomonas mohnii* and *Rhizobium* sp. were detected only in AG, and *Sphingobacterium kitahiroshimense* only in CH (Table 1). No fungal species were shared in all samples. *Arthrinium* sp., *Mucor hiemalis*, *O. sinensis*, *Paecilomyces hepiali*, and *Parengyodontium album* were unique for mummified larvae.

The percentages of the microbe species isolated from different media, temperatures and aeration conditions are presented in Supplementary Table 1. Generally, more bacterial and fungal species were isolated from the plates on G5 medium, at 23°C and under normoxia.

### Culture-Independent Communities

#### Microbial Diversity

A total of 1,204,000 high-quality bacterial (16S rRNA) and 1,201,711 high-quality fungal (ITS) clean reads were obtained from 24 samples representing 8 types of samples (AH, AG, BH, BG, CH, CG, DT, and ET) with three replicates for each group and over 99% of the reads met the demand of Q30 (Supplementary Table 2), indicating high quality of sequencing. A total of 230,841 amplicon sequences from the V4 region of the bacterial 16S rRNA gene and 711,904 sequences from fungal ITS were obtained (Supplementary Table 3). The 16S and ITS sequences were clustered into 537 and 218 OTUs, respectively. For bacteria, 222 genera (dominant genera include *Pseudomonas*, *Stenotrophomonas*, *Rhodococcus*) belonging to 20 phyla (dominant phyla included Proteobacteria, Firmicutes, Actinobacteria, Bacteroidetes), 42 classes, 89 orders, and 146 families were identified from 537 OTUs. For fungi, 54 genera (mainly *Ophiocordyceps* and *Verticillium*) belonging to 5 phyla (Ascomycota, Basidiomycota, Mortierellomycota, Mucoromycota, and Chytridiomycota), 17 classes, 43 orders, and 56 families were identified from 218 OTUs (Supplementary Table 3).

From all samples 47 shared bacterial OTUs were detected, with one class, 7 families, 24 genera and 15 species including *Bartonella apis*, *Clostridium* sensu stricto 1, *Helicobacter ganmani*, *Mycoplasma pulmonis*, *Rhodococcus degradans*, and *Rhodococcus hoagie* (Supplementary Table 4 and Supplementary Figure 1). The unique bacterial OTUs were present in all the samples, but their abundances were very low. 15 shared fungal OTUs were found, including Pseudeurotiaceae and Mortierellaceae at family level; *Chaetomium*, *Penicillium*, and *Pezicula* at genus level; and *Fusarium oxysporum*, *Mortierella humilis*, *O. sinensis*, and *Verticillium leptobactrum* at species level. Only three unique fungal OTUs (Agaricomycetes, *Oidiodendron*, and *Candida*) from BG, two OTUs (*Cenococcum* and *Ophiostoma*) from CG, and one OTU (Sordariomycetes) from ET were identified. These bacterial and fungal OTUs in all samples were considered as shared species in the larvae, whether in the hemolymph or gut, and with or without *O. sinensis*. Beside the shared OTUs, AG and BG samples shared the most bacterial and fungal OTUs among all samples (Supplementary Figure 1), indicating the microbial OTU structure similarity between AG and BG.

**FIGURE 2:**
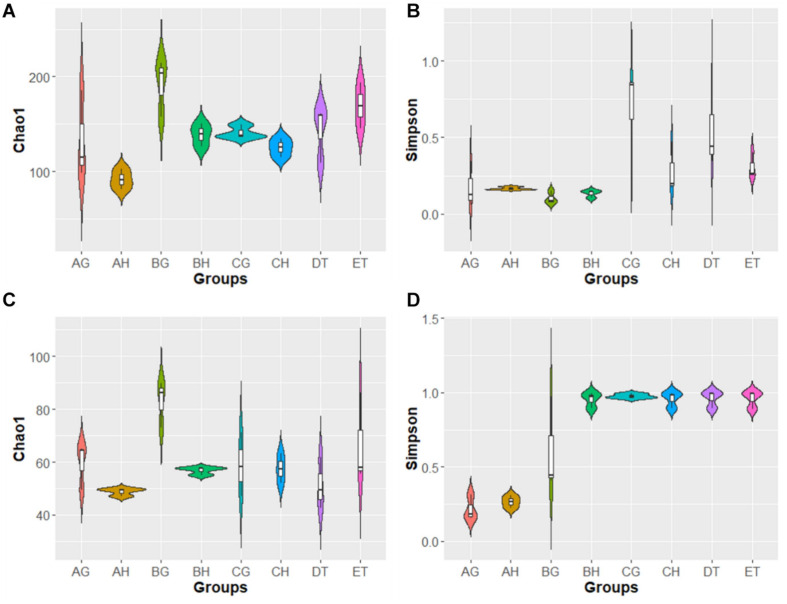
Violin plot of Chao1 and Simpson index of bacterial **(A,B)** and fungal **(C,D)** operational taxonomic units (OTUs) among eight samples. AH, BH, and CH were represented as hemolymph samples respectively from sample A (uninjected living larvae), B (living larvae with high blastospore load) and C (freshly mummifying larvae without a mycelial coating); AG, BG, and CG were represented as gut samples respectively also from samples A–C; DT and ET indicated tissue samples from sample D (mummified larvae with mycelial coating) and E (completely mummified larvae with mycelia).

In the alpha-diversity analysis (Figure 2 and Supplementary Table 5), compared with stages AH and AG, slightly higher bacterial diversities but markedly lower fungal diversity (as indicated by the Simpson index) were detected in BH and BG; bacterial and fungal diversity decreased when the larvae became mummified. Bacterial abundance (as indicated by the Chao1 index) increased and fungal abundance was similar in the larvae injected with blastospores. These results are consistent with rank-abundance curves (Supplementary Figure 2). Rarefaction curves for all samples indicated that the sequence depth was reliable for both bacterial and fungal identification (Supplementary Figure 2). In general, high blastospore load increased bacterial diversity but sharply decreased fungal diversity in the larval hemolymph and gut. When the larvae became mummified, the bacterial and fungal diversity decreased.

**FIGURE 3:**
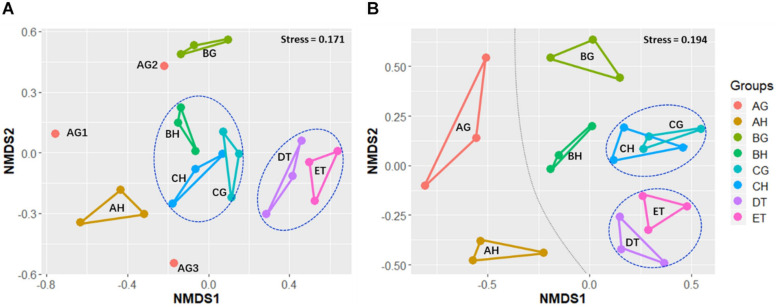
Non-metric multidimensional scaling (NMDS) analysis of beta-diversity based on the Bray–Curtis distance matrix for bacterial **(A)** and fungal **(B)** communities of all samples, with the stress of 0.171 and 0.194, respectively. AH and AG, hemolymph and gut of uninjected living larvae; BH and BG, hemolymph and gut of living larvae with high blastospore load; CH and CG, freshly mummifying larvae without mycelia coating; DT, tissues of the mummified larvae coated with mycelia; ET, tissues of the completely mummified larvae with mycelia.

Non-metric multidimensional scaling was applied to evaluate the beta-diversity changes in the eight sample types, based on the Bray–Curtis distance matrix. For all samples, the bacterial beta-diversities could be divided into four groups. The bacterial communities of ET were significantly different from those of BH (ANOSIM: *R* = 1, *p* = 0.05) and BG (*R* = 1, *p* = 0.05). No significant differences in bacterial communities were found between CH and CG (*R* = 0.59, *p* = 0.12), or DT and ET (*R* = -0.19, *p* = 0.8; Figure 3 and Supplementary Table 6). The fungal beta-diversities could be separated into six groups. The fungal communities of the larvae without *O. sinensis* infection (AH and AG) were significantly different from those of the larvae with *O. sinensis* infection (BH, BG, CH, CG, DT, and ET). There were significant differences of beta-diversities between the samples AG and CG (ANOSIM: *R* = 1, *p* = 0.05), AH and BH (*R* = 1, *p* = 0.06), and AH and CH (*R* = 1, *p* = 0.05). No significant differences were found between CH and CG (*R* = -0.111, *p* = 0.63), AH and AG (*R* = 0.059, *p* = 0.12) or DT and ET (*R* = 0.111, *p* = 0.43). These results indicate again that *O. sinensis* infection reduced fungal diversity (Figure 3 and Supplementary Table 6).

**FIGURE 4:**
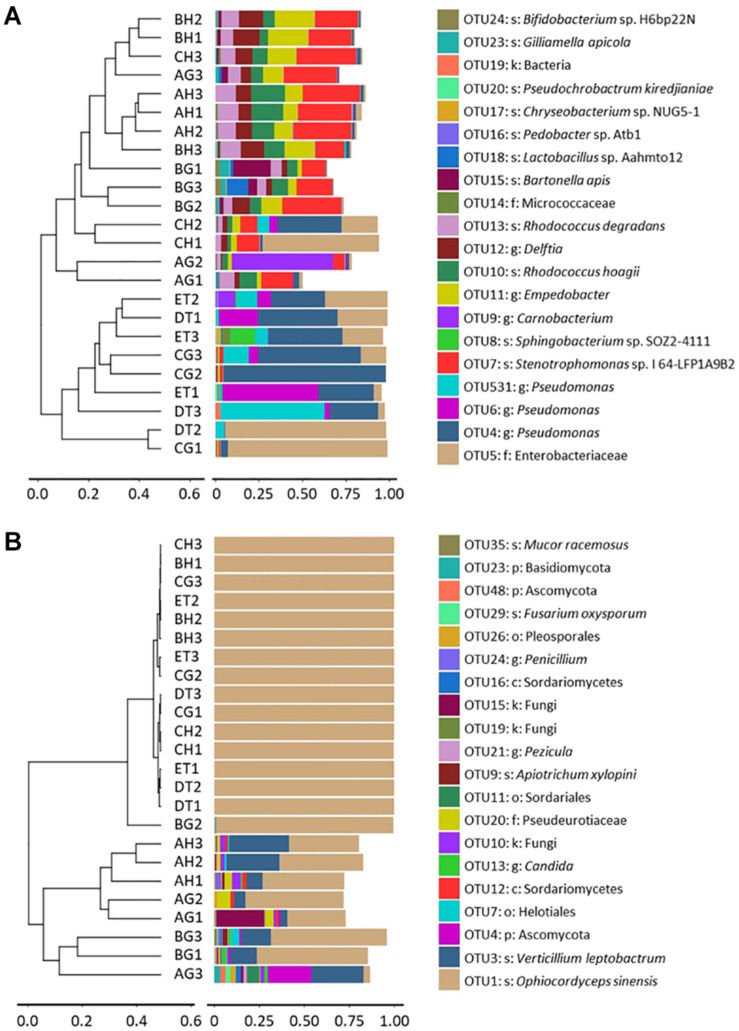
Clustering analysis of beta-diversity based on the Bray–Curtis distance matrix for bacterial **(A)** and fungal **(B)** communities of all samples by the UPGMA method. AH and AG, hemolymph and gut of un-injected living larvae; BH and BG, hemolymph and gut of living larvae with high blastospore load; CH and CG, freshly mummifying larvae without mycelia coating; DT, tissues of the mummified larvae coated with mycelia; ET, tissues of the completely mummified larvae with mycelia.

Clustering analysis was also done to evaluate the beta-diversity changes in the 8 sample types, based on the Bray–Curtis distance matrixes. The bacterial diversities could also be separated into two groups: One group consisted of the samples of BG, CH and CG, DT and ET, which contained Enterobacteriaceae and Pseudomonadaceae, and were affected by the injection and growth of the blastospores; and another group consisted of the samples without *O. sinensis* challenge, or from BH and CH, which contained top OTUs belonging to *Empedobacter* sp., *Delftia* sp., *Sphingobacterium* sp. SOZ2-4111, *R. degradans* and *R. hoagie*. The fungal diversities could be separated into two groups: One group contained the larvae with *O. sinensis* infection, whereas the other grouped the larvae without *O. sinensis* infection. In the former group, over 99% fungal richness belonged to the *O. sinensis* fungus. In the latter group, *V. leptobactrum* became the dominant fungal species (Figure 4).

Based on the significant differences among the samples by using LDA, 156 bacterial OTUs were selected as biomarkers, including 51 from AG, 21 from AH, 47 from BG, 23 from BH, 2 from CG, 3 from CH, 2 from DT and 7 from ET, indicating the high bacterial diversities from the living healthy larvae (AH and AG). 29 of 156 bacterial OTUs were identified to be species level, such as *Stenotrophomonas* sp. (LDA score: 5.12, group: AH), *R. hoagii* (4.84, AH) and *R. degradans* (4.67, AH; Supplementary Table 7). 59 fungal OTUs were selected as biomarkers, including 22 from AG, 18 from AH, 8 from BG, 2 from BH, 2 from CG, and 6 from DT, indicating the high fungal diversities from AG and AH (Supplementary Table 7). 10 of 59 fungal OTUs were identified to be species level, including *V. leptobactrum* (LDA score: 5.082, group: AH), *Mortierella_*sp. (4.328, BG) and *Dothideomycetes_*sp. (4.210, DT).

### Bacterial and Fungal Structures

**FIGURE 5:**
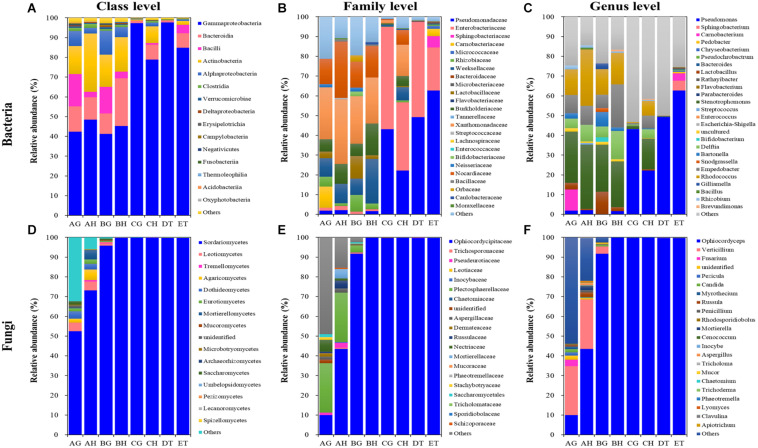
Relative abundances of the top 10 bacterial **(A–C)** and fungal **(D–F)** classes **(A,D)**, families **(B,E)** and genera **(C,F)** in eight types of samples (AG, AH, BG, BH, CG, CH, DT, and ET). “Others” includes classes, families or genera beyond the top 10. AH and AG, hemolymph and guts of un-injected living larvae; BH and BG, hemolymph and guts of living larvae with high blastospore load; CH and CG, freshly mummifying larvae without mycelia coating; DT, tissues of the mummified larvae coated with mycelia; ET, tissues of the completely mummified larvae with mycelia.

A total of 8, 12, 7, 13, 9, and 5 dominant bacterial OTUs (relative abundances > 1%) were identified from AH, AG, BH, BG, CH, and CG, respectively (Supplementary Table 8). One dominant bacterial species, *Stenotrophomonas* sp. I64-LFP1A9B2 was shared among six types of samples. However, the relative abundance of *Stenotrophomonas* sp. I64-LFP1A9B2 was significantly suppressed in the CG group (1.54%), being at least ten times lower than that in the other five types of samples (at least 15.59%). Two dominant bacterial species, *R. hoagii* (> 6.49%) and *R. degradans* (> 5.15%) were shared among non-mummified samples (AG, AH, BG, and BH) and the hemolymph of early mummified larvae (CH). In addition to the shared species above, the guts of non-mummified larvae (AG and BG) shared three more dominant species, including *Bartonella apis* (> 2.84%), *Lactobacillus* sp. Aahmto12 (> 1.22%) and *Gilliamella apicola* (> 1.25%). Three and nine dominant species were found in DT and ET, respectively. The above shared dominant bacterial species were significantly suppressed in the mummified stages (DT and ET), while *Sphingobacterium* sp. SOZ2-4111 (5.05%) was significantly increased in ET. Moreover, 4 dominant bacterial OTUs significantly increased in the mummified stages (CH, CG, DT, and ET), including Enterobacteriaceae (family level) and three *Pseudomonas* sp. (genus level). Top 10 bacterial OTUs from all samples were listed based on the relative abundance (%; Figure 5 and Supplementary Table 8), including 13 at class level; 24 at family level; *Bacillus*, *Bacteroides*, *Bartonella*, *Bifidobacterium*, *Brevundimonas*, *Carnobacterium*, *Chryseobacterium*, *Delftia*, *Empedobacter*, *Enterococcus*, *Escherichia*–*Shigella*, *Flavobacterium*, *Gilliamella*, *Lactobacillus*, *Parabacteroides*, *Pedobacter*, *Pseudochrobactrum*, *Pseudomonas*, *Rathayibacter*, *Rhizobium*, *Rhodococcus*, *Snodgrassella*, *Sphingobacterium*, *Stenotrophomonas*, and *Streptococcus* at genus level, with *Lactobacillus* being shared in all samples. *Pseudomonas* became the dominant bacterial genus (Figure 5 and Supplementary Tables 8, 9). The dominant bacterial OTUs at species level included *Stenotrophomonas* sp. I 64-LFP1A9B2 from all samples; *R. hoagii* and *R. degradans* from AH, AG, BH, BG, and CH; *Bartonella apis* from AG, BH, and BG; *Gilliamella apicola* from AG and BG; and *Lactobacillus* sp. Aahmto12 from AG and BG; *Bifidobacterium* sp. H6bp22N and *Apibacter* sp. wkB309 from BG (Figure 5 and Supplementary Tables 8, 9).

A total of 9, 18, and 2 dominant fungal OTUs were identified from the AH, AG and BG samples, respectively. Two dominant fungal species were shared among these samples, including *O. sinensis* (9.96, 43.41, and 91.69%) and *V. leptobactrum* (24.89, 25.03, and 3.49%). *O. sinensis* was the only dominant OTU among the BH, CG, CH, DT, and ET samples, covering more than 91% relative abundance. *O. sinensis* was abundant in all samples (9.96–99.75%), including the gut (9.96%) and hemolymph (43.41%) samples without *O. sinensis* infection (AG and AH), implying the potential inherent symbiosis between the larvae of *T. xiaojinensis* and the fungus *O. sinensis*. Meanwhile, *O. sinensis* was mainly present in the hemolymph rather than in the gut under uninfected conditions. *V. leptobactrum* was another dominant fungal species in the gut (24.89%) and hemolymph (25.03%) under normal conditions, and decreased sharply after *O. sinensis* infection (Figure 5 and Supplementary Table 8). Top 10 fungal OTUs from all samples were ranked based on their relative abundance (%; Figure 5), including 16 at class level; 20 at family level; *Apiotrichum*, *Aspergillus*, *Candida*, *Cenococcum*, *Chaetomium*, *Clavulina*, *Fusarium*, *Inocybe*, *Lyomyces*, *Mortierella*, *Mucor*, *Myrothecium*, *Ophiocordyceps*, *Penicillium*, *Pezicula*, *Phaeotremella*, *Rhodosporidiobolus*, *Russula*, *Trichoderma*, *Tricholoma*, and *Verticillium* at genus level, with *Ophiocordyceps*, *Penicillium*, and *Verticillium* being common in all samples. High fungal diversities were detected from the hemolymph (AH) and guts (AG) of the larvae without *O. sinensis* infection, where *V. leptobactrum*, *O*. *sinensis*, *F. oxysporum*, and *M. verrucaria* were identified at species level, with *V. leptobactrum* and *O. sinensis* being the dominant species in AH and AG. Interestingly, *O*. *sinensis* sequences were detected from uninjected larvae, although they were only 1/435 of those detected from BH. For the analysis of fungal community structures, the richness of *O. sinensis* in the gut of the injected larvae (BG) was much higher than that in the hemolymph (BH; Supplementary Table 5). When the larvae became mummified, *O*. *sinensis* was the dominant fungal species (> 99.5% relative abundance) in both the larval hemolymph and gut (CH and CG), as well as in the larval tissues (DT and ET), whereas other fungi were seriously inhibited (Supplementary Tables 8, 9).

## Discussion

During artificial cultivation of the Chinese cordyceps, the *T. xiaojinensis* host larvae were fed on the roots of *P. anserina* collected from the wild, which allowed the larval microbiota to establish. Using culture-dependent as well as culture-independent methods, the microbiota in the hemolymph and gut of *T. xiaojinensis* larvae was compared among uninjected living larvae, injected living larvae containing blastospores, and freshly mummifying larvae without mycelia coating. In addition, the tissues of larvae from mummified larvae coated with mycelia and completely mummified larvae with mycelia were also analyzed. The discovery of many opportunistic pathogenic bacteria in the hemolymph of the living larvae without *O. sinensis* infection, the high larval microbial diversities influenced by *O. sinensis* infection and the involvement of dominant bacteria during the mummification process of infected larvae provide new insight into the infection and mummification mechanism of *O. sinensis* in its *Thitarodes* hosts.

### Microbiota in Insect Hemolymph and Gut Without *O. sinensis* Infection

Hemolymph is recognized as a key mediator of nutritional and immunological homeostasis in insects and is generally considered to be microbe-free, or nearly so, in healthy insects (Lemaitre and Hoffmann, 2007). Now, overwhelming evidence indicates that various non-pathogenic microorganisms can stably or transiently inhabit hemolymph in a diversity of insects (Blow and Douglas, 2019). The most reported hemolymph microorganisms are bacteria of the genus *Spiroplasma* (Phylum Tenericutes, Family Mollicutes) widely associated with insects in the Hymenoptera, Diptera, Lepidoptera, Hemiptera and Coleoptera orders (Clark, 1982), with reported densities of ca. 10^8^ mL^–1^ hemolymph in *Drosophila melanogaster* (Herren et al., 2014). A second group of bacteria found abundantly in the insect hemolymph are members of the Enterobacteriaceae (γ-proteobacteria) in aphids, specifically *Serratia symbiotica* and the sister taxa *Hamiltonella defensa* and *Regiella insecticola* (Henry et al., 2013; Zytynska et al., 2016). Surprisingly, in the present study, from the hemolymph of healthy, laboratory-cultured *T. xiaojinensis* larvae (i.e., without *O. sinensis* infection), 14 bacterial species in 12 genera including entomopathogenic *Serratia* spp. and *Pseudomonas* spp. were isolated, using a culture-dependent method (Table 1). Further, 164 bacterial OTUs including the dominant species (relative abundance > 1%) *Stenotrophomonas* sp. I 64-LFP1A9B2 (32.46%), *R. hoagii* (17.07%) and *R. degradans* (11.39%), and 73 fungal OTUs including the dominant species *M. humilis* (1.07%), *O. sinensis* (43.41%) and *V. leptobactrum* (25.03%) were identified by a culture-independent method. These findings suggest that these bacteria and fungi, which are totally different from those reported from other insects (for example, *D. melanogaster* and aphids; Henry et al., 2013; Herren et al., 2014; Zytynska et al., 2016), may utilize host nutritional resources, and persist in the hemolymph by a combination of evasion and tolerance of insect immune effectors. To the best of our knowledge, this is the first study to isolate such a high number of pathogenic bacterial species from the hemolymph of healthy insect larvae. How these microorganisms can survive in the larval hemolymph of *T. xiaojinensis* and succeed in evading the insect’s immune system are questions for further study.

Without *O. sinensis* injection, the gut of healthy laboratory-reared larvae contained 14 bacterial species in 11 genera and 3 fungal species (*Apiotrichum porosum*, *Phialemonium inflatum*, and *Sterigmatomyces halophilus*) in 3 genera based on the culture-dependent method (Table 1); and 259 bacterial OTUs including dominant species *Stenotrophomonas* sp. I 64-LFP1A9B2 (25.34%), *R. hoagie* (6.49%), *R. degradans* (6.35%), *Bartonella apis* (2.84%), *Lactobacillus* sp. Aahmto12 (1.22%) and *Gilliamella apicola* (1.25%), and 108 fungal OTUs including *F. oxysporum* (2.23%), *Myrothecium verrucaria* (1.37%), *O. sinensis* (9.96%), and *V. leptobactrum* (24.89%), based on the culture-independent method. Compared with eight bacterial genera (*Staphylococcus*, *Bacillus*, *Klebsiella*, *Pseudomonas*, *Aeromonas*, *Plesiomonas*, *Sporosarcina*, and *Neisseria*) isolated by Zhuo et al. (2004), and eight bacterial genera (*Enterobacter, Carnobacterium*, *Novosphingobium*, *Acinetobacter*, *Pseudomonas*, *Klebsiella, Pantoea, and Delftia*) by Liu et al. (2008) from the guts of *T. gonggaensis* larvae collected in the wild, more bacterial genera (14 vs. 8) were found in the present study by a culture-dependent method. Only *Pseudomonas* and *Carnobacterium* were common in wild *T. gonggaensis* larvae and laboratory-reared *T. xiaojinensis* larvae, which may be due to differences in the microbiota of wild and laboratory insect populations, different *Thitarodes* species and microbe detection methods.

Few *O. sinensis* sequences (1/435 of those detected from BH) were detected by a culture-independent method in both the hemolymph and gut of healthy larvae, i.e., without fungal infection. The roots of *P. anserina* may be associated with the *O. sinensis* fungus as a plant endophyte (Zhong et al., 2014; Lei et al., 2015; Wang et al., 2020). Whether few fungal spores were introduced into the larvae from the larval food (i.e., roots of *P. anserina* collected in the wild) needs further study.

### Microbiota in Insect Hemolymph and Gut With *O. sinensis* Infection

The microbiota of the insect gut plays crucial roles in modulating the interactions between the insect host and intestinal pathogens. The infection of the Chinese white wax scale insect *Ericerus pela* by *Cladosporium langeronii* and *C. sphaerospermum* fungi had little effect on the fungal community but strongly influenced the bacterial community (Sun et al., 2008). The pathogenic fungus *Beauveria bassiana* interacts with the gut microbiota of mosquitoes to accelerate host mortality, and fungal infection causes dysbiosis of mosquito gut microbiota with a significant increase in gut bacterial load and a significant decrease in bacterial diversity (Wei et al., 2017). Contrary to Wei et al. (2017), here growth of *O. sinensis* blastospores in the hemolymph of live larvae greatly inhibited the culturable bacterial community in the hemolymph but stimulated the culturable bacterial and fungal community in the gut; when the living larvae contained a high load of blastospores, nine culturable bacterial species disappeared from the hemolymph, but three more bacterial species and three-times more fungal species were isolated from the gut, compared with the larvae not containing blastospores.

**FIGURE 6:**
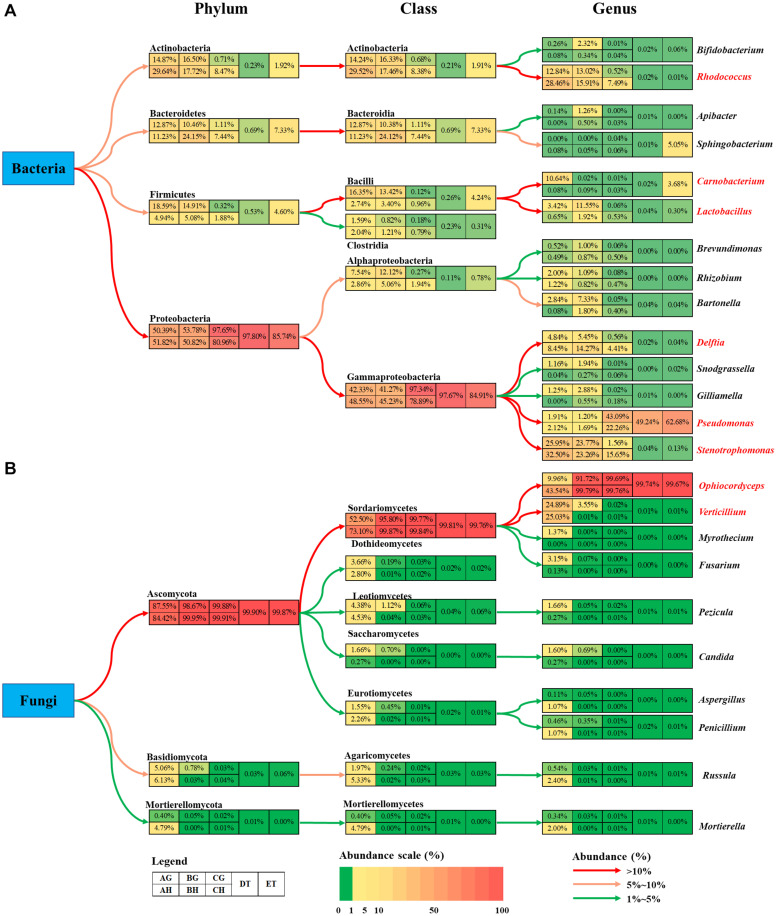
Summary of the changes in bacteria **(A)** and fungi **(B)** community among eight types of samples. The colored boxes represent corresponding samples according to the legend. Relative abundance is indicated by color gradients; the color from green through yellow to red represents the value from low to high. Colored arrows show the abundance changes from higher taxonomic level to lower level. AH and AG, hemolymph and gut of un-injected living larvae; BH and BG, hemolymph and gut of living larvae with a high blastospore load; CH and CG, freshly mummifying larvae without mycelia coating; DT, tissues of the mummified larvae coated with mycelia; ET, tissues of the completely mummified larvae with mycelia.

The bacterial abundance of *Carnobacterium* and *Lactobacillus* species in the gut was much higher than that in the hemolymph (Figure 6A). *O. sinensis* was the most abundant fungal species in *T. xiaojinensis*, both in the hemolymph and the gut, and in both mummified stages of the larvae. Except for *Ophiocordyceps* species, most of the fungal species were significantly suppressed in the living larvae containing high numbers of blastospores. In uninfected larvae, *Verticillium* species was evenly distributed in the gut and hemolymph with a high relative abundance, while *Myrothecium*, *Fusarium*, *Pezicula*, and *Candida* were mainly distributed in the gut, and *Ophiocordyceps*, *Aspergillus*, *Penicillium*, *Russula*, and *Mortierella* were dominant in the hemolymph (Figure 6B). Based on the OTU diversity of unculturable bacteria and fungi, in general, high blastospore load increased bacterial diversity but sharply decreased fungal diversity in the larval hemolymph and gut. When the larvae became mummified, the bacterial and fungal diversity declined.

### Microbiota Comparison of Culture-Dependent and -Independent Methods

The culture-independent method detected more OTUs than the culture-dependent method in this study. Eight (*Carnobacterium*, *Chryseobacterium*, *Flavobacterium*, *Pseudomonas*, *Rhizobium*, *Rhodococcus*, *Sphingobacterium* and *Stenotrophomonas*) of 16 culturable bacterial genera and 4 (*Apiotrichum*, *Mucor*, *Ophiocordyceps* and *Penicillium*) of 13 cultural fungal genera were included in the top 10 OTU genera for all samples. *Ophiocordyceps* and *Pseudomonas* were dominant genera in both detection methods, indicating the overlapping detection of key microbes by both methods. In contrast, Zhang et al. (2010) reported that very few fungal OTUs from the naturally occurring Chinese cordyceps were shared by culture-dependent and -independent methods.

Based on OTUs, the dominant bacterial species *Stenotrophomonas* sp. I 64-LFP1A9B2, *R. hoagii* and *R. degradans* and fungal species *O. sinensis* and *V. leptobactrum* were shared in the hemolymph and gut of uninjected larvae. However, with the exception of *O. sinensis*, these microbe species were not isolated from the media plates, even when using different media (LB, HIA, G5, and GSA) in the incubators with or without aeration at 13 and 23°C, respectively. *V. leptobactrum* is a rare rock-inhabiting fungal species from serpentinic rocks playing an important role in the bioweathering (Daghino et al., 2009). A specific medium (Daghino et al., 2009) was used to isolate this fungus but this attempt was not successful. Due to its dominant occurrence in the hemolymph and gut of uninjected larvae and its shared presence in all samples, *V. leptobactrum* may be a representative fungus in *Thitarodes* moths. The microbial diversity trend appeared not to be exactly similar for the two detection methods, which may have been influenced by microbial culturability.

### Mummification-Related Bacteria and Fungi

So far, a variety of microbial communities have been identified from naturally occurring Chinese cordyceps and the gut of wild *Thitarodes/Hepialus* larvae, as well as from their microhabitats, by using traditional culture-dependent and culture-independent methods, including PCR-based molecular markers, sequencing of ribosomal DNA and high-throughput sequencing (Zhuo et al., 2004; Liu et al., 2008; Liang et al., 2008, 2019; Yu et al., 2008; Zhang et al., 2009, 2010; Xia et al., 2015, 2016). All these communities may provide the foundation for further discovery of new valuable microorganism resources for the medicine industry. However, no information is available on the direct effects of the microbiota on the regulation of artificial production of Chinese cordyceps.

Cultivation of Chinese cordyceps at commercial scale has been successfully established (Li et al., 2016, 2019; Han et al., 2019; Liu et al., 2020). However, several factors such as the degeneration of the fungus, high mortality of host larvae by pathogens, low and slow infection and mummification rate constrained the efficient production of Chinese cordyceps (Zhou et al., 2013; Lu et al., 2015; Qin et al., 2018). Low and slow mummification rate was the most important uncontrolled factor during the cultivation process (Liu et al., 2019). The microbiota in the hemolymph and gut may be involved in this process. For example, the opportunistic pathogenic bacterium *Serratia marcescens* overgrows in the midgut of *Anopheles stephensi* and translocates to the hemocoel, which promotes *B. bassiana* killing the mosquitoes (Wei et al., 2017). In the present study, four culturable bacterial species (*Chryseobacterium* sp., *P. fragi*, *S. plymuthica*, *S. proteamaculans*) were common in freshly or completely mummified larvae and actively overgrew. *F. frigidimaris* and *P. poae* were detected only in freshly and/or completely mummified larvae and *Arthrinium* sp. was the only culturable fungal species from freshly mummified larvae, indicating their high loads at this stage of infection. *O*. *sinensis* and *Pseudomonas* spp. became dominant microbe species, when the infected larvae became mummified. *Pseudomonas* spp. are regarded as psychrotolerant and broad-host-range entomopathogenic bacteria exhibiting insecticidal activity toward certain agricultural pests (Chen et al., 2014; Mei et al., 2016). It appears that these bacteria and fungi might be involved in the blastospore-mycelium transition during larval mummification process. Whether blastospores induce the larval immune system to inhibit the bacterial and fungal growth in the hemolymph and how the blastospore-mycelium transition destroys the larval immune system allowing bacterial outbreak warrants further study. Furthermore, as *Chryseobacterium* sp., *P. fragi*, *S. plymuthica*, and *S. proteamaculans* may be opportunistic pathogens for human beings (Kertesz and Thai, 2018), the possible presence of these bacteria in the fresh Chinese cordyceps should be carefully considered.

Interestingly, *O. sinensis* was detected only in the hemolymph of freshly mummified larvae. This phenomenon was also observed in the routine practice in the laboratory (unpublished data). It appears that not all blastospores from the hemolymph can survive well *in vitro* when they are spread onto the media plates. Possibly, the blastospores exhibit different phases in different environments to adapt growth *in vivo* and *in vitro*. *Paecilomyces hepiali* and *Isaria farinosa* were considered to be important entomopathogenic fungi for *Thitarodes* larvae during the artificial cultivation of Chinese cordyceps (Han et al., 2019). However, *I. farinosa* was not abundant in the culture plates. Perhaps *I. farinosa* is an opportunistic fungus for the moth larvae. It should be pointed out that some minor bacterial or fungal species might not have been detected from the plates under the present experimental conditions, although five media, low and temperate temperatures, and normoxia and anoxia conditions were used for the detection of microbiota in this study.

## Conclusion

The composition of the hemolymph and gut microbiota from laboratory-reared *T. xiaojinensis* larvae with or without injected *O. sinensis* blastospores is unique. The uninfected larval hemolymph contained as many culturable bacterial species as did the larval gut. *Stenotrophomonas* sp. I 64-LFP1A9B2, *R. hoagii*, *R. degradans*, *O. sinensis*, and *V. leptobactrum* OTU species were dominant and shared in the hemolymph and gut of uninjected larvae. The growth of blastospores sharply inhibited the culturable bacterial community in the hemolymph but greatly enhanced the bacterial and fungal community in gut of live larvae. However, high blastospore load increased bacterial diversity but sharply decreased fungal diversity in the larval hemolymph and gut based on OTUs. Four culturable bacterial species (*Chryseobacterium* sp., *P. fragi*, *S. plymuthica*, *S. proteamaculans*) overgrew and *O*. *sinensis* and *Pseudomonas* spp. became dominant microorganisms, when the infected larvae became mummified, indicating their possible involvement in the larval mummification process.

## Data Availability Statement

The datasets generated in this study can be found in online repositories. The names of the repository/repositories and accession number(s) can be found in the article/Supplementary Material.

## Ethics Statement

No special permits were required for sampling of organisms in this study. All samples were collected by researchers with introduction letters from the Guangdong Institute of Applied Biological Resources and with the help of local herdsmen.

## Author Contributions

R-CH and PD designed and coordinated the research. HW and LC collected the samples. HW conducted the research. Z-CR and HW analyzed the data. R-CH, PD, HW, and Z-CR wrote the manuscript. All authors approved the final version of the manuscript.

## Conflict of Interest

The authors declare that the research was conducted in the absence of any commercial or financial relationships that could be construed as a potential conflict of interest.

## Footnotes

1 http://www.ncbi.nlm.nih.gov/genbank/

2 http://www.arb-silva.de

3 http://unite.ut.ee/index.php
