# Supplementary material for: Infection of Ophiocordyceps sinensis Fungus Causes Dramatic Changes in the Microbiota of Its Thitarodes Host
Source: Front Microbiol. 2020 Dec 3;11:577268. doi: 10.3389/fmicb.2020.577268 (PMC7744566; doi:10.3389/fmicb.2020.577268)
Supplement: Supplementary file 1 [file Data_Sheet_1.docx]

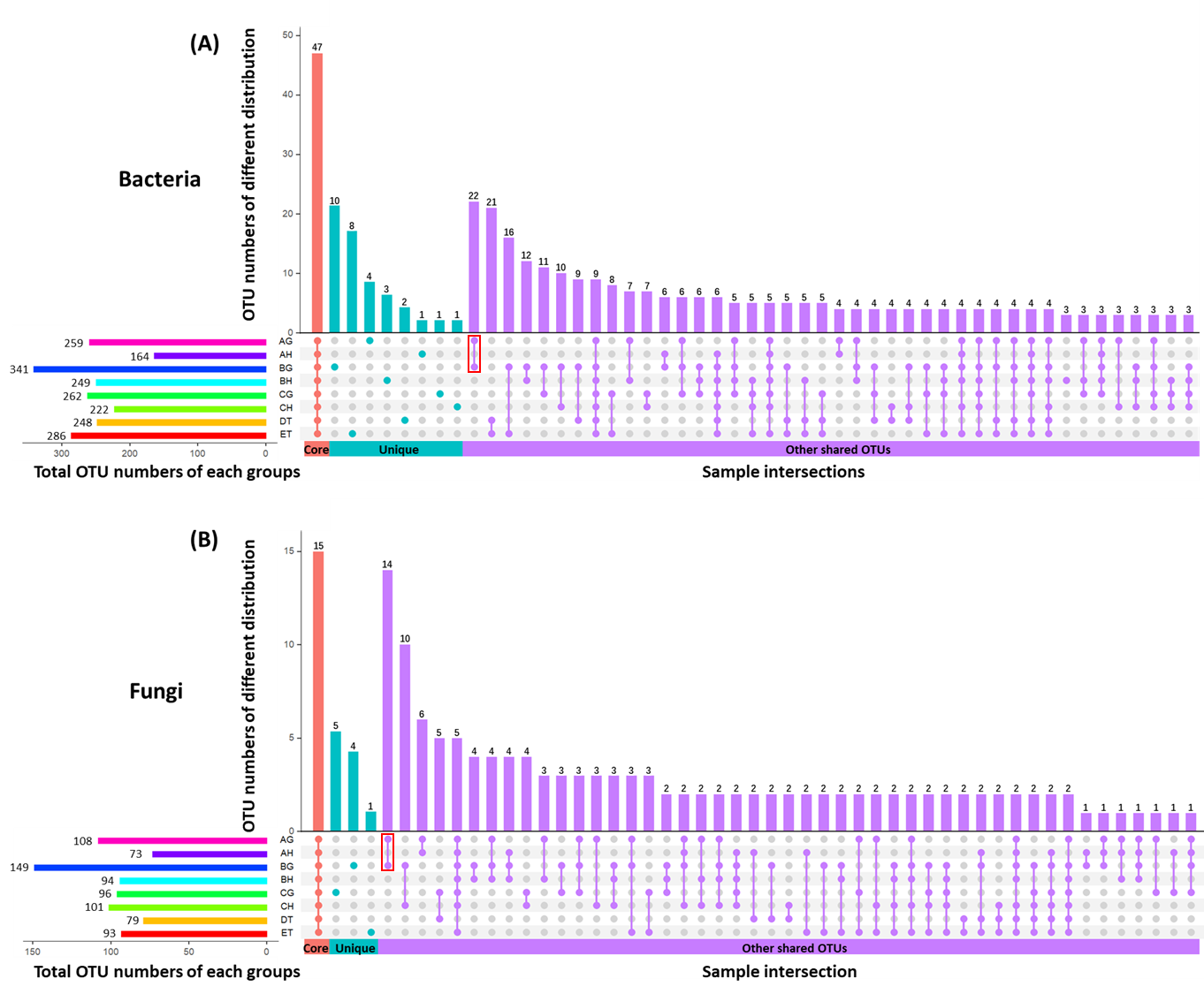


**SUPPLEMENTARY FIGURE 1.** UpSetR plot depicting the unique and shared bacterial (A) and fungal (B) OTUs among 8 samples. Horizontal bars represent the total OTU numbers of each group, vertical bars represent the Core, Unique and other shared OTU numbers of different sample intersections. The shared OTUs between AG and BG are marked with a red box. AH and AG, hemolymph and gut of un-injected living larvae; BH and BG, hemolymph and gut of living larvae with high load of blastospores; CH and CG, freshly mummifying larvae without mycelia coating; DT, tissues of the mummified larvae coated with mycelia; ET, tissues of the completely mummified larvae with mycelia.
