# Supplementary material for: Infection of Ophiocordyceps sinensis Fungus Causes Dramatic Changes in the Microbiota of Its Thitarodes Host
Source: Front Microbiol. 2020 Dec 3;11:577268. doi: 10.3389/fmicb.2020.577268 (PMC7744566; doi:10.3389/fmicb.2020.577268)
Supplement: Supplementary file 2 [file Data_Sheet_2.docx]

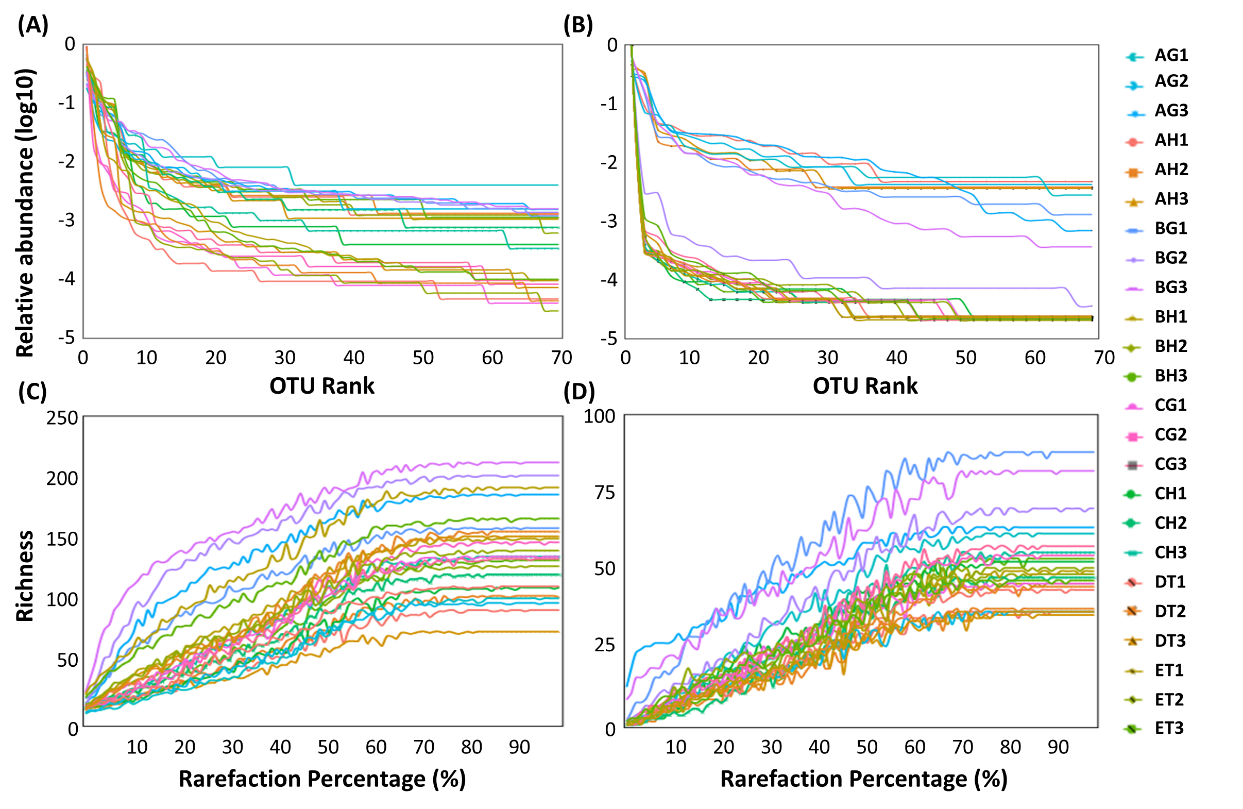


**SUPPLEMENTARY FIGURE 2.** Rank-abundance and rarefaction curves for all samples. Rank-abundance curves for bacteria (A) and fungi (B), Rarefaction curves for bacteria (C) and fungi (D).
