# Supplementary material for: Infection of Ophiocordyceps sinensis Fungus Causes Dramatic Changes in the Microbiota of Its Thitarodes Host
Source: Front Microbiol. 2020 Dec 3;11:577268. doi: 10.3389/fmicb.2020.577268 (PMC7744566; doi:10.3389/fmicb.2020.577268)
Supplement: Supplementary file 3 [file Table_1.docx]

**Supplementary Table 1.** The percentages of the microbe species isolated from different media (A), different temperatures (B) and normoxic or anoxic condition (C)

**A**

| Media^3^ | Percentage of the isolated microbe species from different samples^1^ | | | | |
| --- | --- | --- | --- | --- | --- |
|  | A^2^ | B | C | D | E |
| LB | 47.62 | 55.17 | 40.00 | 41.67 | 50.00 |
| HIA | 66.67 | 55.17 | 46.67 | 41.67 | 50.00 |
| G5 | 52.38 | 65.52 | 60.00 | 66.67 | 66.67 |
| GSA | 38.10 | 44.83 | 53.33 | 50.00 | 66.67 |

Notes:

^1^ Percentage of the isolated microbe species from each medium in each sample = The number of bacterial and fungal species isolated from the medium/total number of the isolated microbe species under all conditions (medium, aeration, temperature).

^2^A, hemolymph and guts of living larvae without injected fungi; B, living larvae with high load of blastospores; C, mummifying larvae without mycelia coating; D, mummified larvae coated with mycelia; E, stiffly mummified larvae with mycelia.

^3^Media: LB; HIA; G5; GSA.

**B**

| Temperature (°C) | Percentage of the isolated microbe species from different samples^1^ | | | | |
| --- | --- | --- | --- | --- | --- |
|  | A^2^ | B | C | D | E |
| 13 | 52.38 | 68.97 | 80.00 | 58.33 | 66.67 |
| 23 | 90.48 | 72.41 | 80.00 | 91.67 | 91.67 |

Notes:

^1^Percentage of the isolated microbe species at each temperature in each sample = The number of bacterial and fungal species isolated at the given temperature/total number of the isolated microbe species under all conditions (medium, aeration, temperature).

^2^A, hemolymph and guts of living larvae without injected fungi; B, living larvae with high load of blastospores; C, mummifying larvae without mycelia coating; D, mummified larvae coated with mycelia; E, stiffly mummified larvae with mycelia.

**C**

| Aeration condition | Percentage of the isolated microbe species from different samples^1^ | | | | |
| --- | --- | --- | --- | --- | --- |
|  | A^2^ | B | C | D | E |
| Normoxia | 71.43 | 86.21 | 80.00 | 83.33 | 100.00 |
| Anoxia | 76.19 | 68.97 | 66.67 | 58.33 | 66.67 |

Notes:

^1^ Percentage of the isolated microbe species under normoxic or anoxic condition in each sample = The number of bacterial and fungal species isolated at either normoxic or anoxic condition /total number of the isolated microbe species under all conditions (medium, aeration, temperature).

^2^A, haemolymph and guts of living larvae without injected fungi; B, living larvae with high load of blastospores; C, mummifying larvae without mycelia coating; D, mummified larvae coated with mycelia; E, stiffly mummified larvae with mycelia.
