# Supplementary material for: Infection of Ophiocordyceps sinensis Fungus Causes Dramatic Changes in the Microbiota of Its Thitarodes Host
Source: Front Microbiol. 2020 Dec 3;11:577268. doi: 10.3389/fmicb.2020.577268 (PMC7744566; doi:10.3389/fmicb.2020.577268)
Supplement: Supplementary file 10 [file Table_8.docx]

**Supplementary Table 8. Dominant bacterial (A) and fungal (B) OTUs (Richness abundance>1) in 8 types of samples**

**A**

| **OTU ID** | **AG** | **AH** | **BG** | **BH** | **CG** | **CH** | **DT** | **ET** | **Taxonomic rank** |
| --- | --- | --- | --- | --- | --- | --- | --- | --- | --- |
| OTU_5 | 0.75% | 1.88% | 0.52% | 0.66% | 51.79% | 34.25% | 48.02% | 21.69% | f: Enterobacteriaceae |
| OTU_14 | 0.20% | 0.53% | 0.11% | 0.21% | 0.05% | 0.35% | 0.12% | 1.46% | f: Micrococcaceae |
| OTU_4 | 1.07% | 1.31% | 0.31% | 1.12% | 38.61% | 16.95% | 23.71% | 34.80% | g: *Pseudomonas* |
| OTU_11 | 9.22% | 9.68% | 6.73% | 21.82% | 0.76% | 6.43% | 0.01% | 0.01% | g: *Empedobacter* |
| OTU_12 | 4.84% | 8.45% | 5.45% | 14.27% | 0.56% | 4.41% | 0.02% | 0.04% | g: *Delftia* |
| OTU_6 | 0.38% | 0.29% | 0.10% | 0.11% | 1.20% | 2.07% | 8.93% | 21.07% | g: *Pseudomonas* |
| OTU_531 | 0.20% | 0.41% | 0.08% | 0.41% | 3.26% | 3.24% | 16.60% | 6.80% | g: *Pseudomonas* |
| OTU_9 | 10.64% | 0.08% | 0.02% | 0.09% | 0.01% | 0.03% | 0.02% | 3.68% | g: *Carnobacterium* |
| OTU_763 | 1.01% | 1.18% | 0.54% | 0.78% | 0.06% | 0.47% | 0.00% | 0.00% | g: *Allorhizobium*… |
| OTU_30 | 1.16% | 0.04% | 1.94% | 0.27% | 0.01% | 0.06% | 0.00% | 0.02% | g: *Snodgrassella* |
| OTU_31 | 0.41% | 0.04% | 1.02% | 0.46% | 0.01% | 0.03% | 0.00% | 0.00% | g: *Lactobacillus* |
| OTU_37 | 0.17% | 0.00% | 1.00% | 0.18% | 0.00% | 0.01% | 0.00% | 0.02% | g: *Lactobacillus* |
| OTU_7 | 25.34% | 32.46% | 23.66% | 23.23% | 1.54% | 15.59% | 0.03% | 0.02% | s: *Stenotrophomonas sp.* I 64-LFP1A9B2 |
| OTU_10 | 6.49% | 17.07% | 7.87% | 6.98% | 0.24% | 3.68% | 0.00% | 0.01% | s: *Rhodococcus hoagii* |
| OTU_13 | 6.35% | 11.39% | 5.15% | 8.94% | 0.28% | 3.81% | 0.02% | 0.01% | s: *Rhodococcus degradans* |
| OTU_15 | 2.84% | 0.08% | 7.33% | 1.80% | 0.05% | 0.40% | 0.04% | 0.04% | s: *Bartonella apis* |
| OTU_18 | 1.22% | 0.08% | 6.32% | 0.23% | 0.00% | 0.18% | 0.01% | 0.19% | s: *Lactobacillus sp*. Aahmto12 |
| OTU_8 | 0.00% | 0.08% | 0.00% | 0.05% | 0.03% | 0.04% | 0.01% | 5.05% | s: *Sphingobacterium sp.* SOZ2-4111 |
| OTU_23 | 1.25% | 0.00% | 2.88% | 0.55% | 0.02% | 0.18% | 0.01% | 0.00% | s: *Gilliamella apicola* |
| OTU_24 | 0.26% | 0.08% | 2.28% | 0.34% | 0.01% | 0.04% | 0.00% | 0.02% | s: *Bifidobacterium sp.* H6bp22N |
| OTU_43 | 0.14% | 0.00% | 1.26% | 0.50% | 0.00% | 0.03% | 0.00% | 0.00% | s: *Apibacter sp.* wkB309 |
| other | 26.04% | 14.86% | 25.43% | 17.01% | 1.50% | 7.75% | 2.46% | 5.05% |  |

**B**

| **OTU ID** | **AG** | **AH** | **BG** | **BH** | **CG** | **CH** | **DT** | **ET** | **Taxonomic rank** |
| --- | --- | --- | --- | --- | --- | --- | --- | --- | --- |
| OTU_10 | 1.97% | 3.20% | 0.04% | 0.00% | 0.03% | 0.02% | 0.02% | 0.01% | k: Fungi |
| OTU_15 | 3.75% | 0.40% | 0.01% | 0.00% | 0.00% | 0.00% | 0.00% | 0.00% | k: Fungi |
| OTU_48 | 2.55% | 0.00% | 0.03% | 0.00% | 0.00% | 0.00% | 0.00% | 0.00% | p: Ascomycota |
| OTU_4 | 20.17% | 0.00% | 0.29% | 0.00% | 0.00% | 0.00% | 0.00% | 0.01% | p: Ascomycota |
| OTU_23 | 2.63% | 0.00% | 0.01% | 0.00% | 0.00% | 0.00% | 0.00% | 0.00% | p: Basidiomycota |
| OTU_12 | 0.37% | 1.20% | 0.05% | 0.03% | 0.04% | 0.04% | 0.03% | 0.04% | c: Sordariomycetes |
| OTU_16 | 2.40% | 0.00% | 0.15% | 0.00% | 0.00% | 0.00% | 0.00% | 0.00% | c: Sordariomycetes |
| OTU_30 | 1.00% | 0.13% | 0.12% | 0.00% | 0.00% | 0.00% | 0.00% | 0.00% | o: Helotiales |
| OTU_26 | 2.63% | 0.40% | 0.03% | 0.00% | 0.00% | 0.00% | 0.00% | 0.01% | o: Pleosporales |
| OTU_11 | 5.84% | 0.00% | 0.05% | 0.00% | 0.00% | 0.00% | 0.00% | 0.00% | o: Sordariales |
| OTU_50 | 0.06% | 2.80% | 0.02% | 0.00% | 0.01% | 0.00% | 0.00% | 0.00% | f: Mortierellaceae |
| OTU_37 | 1.03% | 0.00% | 0.00% | 0.00% | 0.00% | 0.00% | 0.00% | 0.00% | f: Nectriaceae |
| OTU_36 | 1.06% | 0.00% | 0.01% | 0.00% | 0.00% | 0.00% | 0.00% | 0.00% | f: Nectriaceae |
| OTU_20 | 1.32% | 2.53% | 0.08% | 0.03% | 0.02% | 0.01% | 0.02% | 0.02% | f: Pseudeurotiaceae |
| OTU_75 | 0.20% | 1.46% | 0.00% | 0.00% | 0.00% | 0.00% | 0.00% | 0.00% | f: Russulaceae |
| OTU_13 | 1.60% | 0.27% | 0.54% | 0.00% | 0.00% | 0.00% | 0.00% | 0.00% | g: *Candida* |
| OTU_21 | 1.66% | 0.27% | 0.05% | 0.00% | 0.02% | 0.01% | 0.01% | 0.01% | g: *Pezicula* |
| OTU_73 | 0.17% | 2.00% | 0.01% | 0.01% | 0.00% | 0.00% | 0.00% | 0.00% | g: *Russula* |
| OTU_29 | 2.23% | 0.13% | 0.07% | 0.00% | 0.00% | 0.00% | 0.00% | 0.00% | s: *Fusarium oxysporum* |
| OTU_72 | 0.06% | 1.07% | 0.01% | 0.00% | 0.01% | 0.00% | 0.00% | 0.00% | s: *Mortierella humilis* |
| OTU_31 | 1.37% | 0.00% | 0.00% | 0.00% | 0.00% | 0.00% | 0.00% | 0.00% | s: *Myrothecium verrucaria* |
| OTU_1 | 9.96% | 43.41% | 91.69% | 99.75% | 99.67% | 99.74% | 99.74% | 99.66% | s: *Ophiocordyceps sinensis* |
| OTU_3 | 24.89% | 25.03% | 3.49% | 0.01% | 0.02% | 0.01% | 0.01% | 0.01% | s: *Verticillium leptobactrum* |
| Others | 11.07% | 15.71% | 3.25% | 0.17% | 0.18% | 0.15% | 0.15% | 0.23% |  |

Notes：A, hemolymph and guts of living larvae without injected fungi; B, living larvae with high load of blastospores; C, mummifying larvae without mycelia coating; D, mummified larvae coated with mycelia; E, stiffly mummified larvae with mycelia.
